# Supplementary material for: Common variants in MMP20 at 11q22.2 predispose to 11q deletion and neuroblastoma risk
Source: Nat Commun. 2017 Sep 18;8:569. doi: 10.1038/s41467-017-00408-8 (PMC5603517; doi:10.1038/s41467-017-00408-8)
Supplement: Supplementary file 1 — Supplementary Information [file 41467_2017_408_MOESM1_ESM.pdf]

File Name: Supplementary Information
Description: Supplementary Figures, Supplementary Tables, Supplementary Notes and Supplementary References.

File Name: Supplementary Data 1
Description: Summary of neuroblastoma samples in this study.

File Name: Supplementary Data 2
Description: List of rare variants in *MMP20* identified from sequencing data.

File Name: Peer Review File
Description:

Supplementary information

Supplementary Note 1

In the supplementary note, we highlighted the novel genomic regions of recurrent focal events and candidate genes discovered in this study. Beside the commonly observed MNA, 11q deletion and 1q deletion variations in neuroblastoma (**Supplementary Fig. 1**), focal amplifications of *ALK*, *CCND1*, *LIN28B*, *MDM2* and 19q13.42 observed in our study have been implicated in neuroblastoma previously [1-5]. However, to our knowledge, recurrent focal amplifications of *MYC*, *ZFHX3*, *KRAS*, *RRAS2* and *CYTH1* have not been reported in neuroblastoma primary tumors before. We further confirmed that the amplified genes were significantly overexpressed in neuroblastoma tumors and cell lines by examining gene expression data from 100 primary tumors and 29 cell lines (**Supplementary Table 1**).

1. Focal amplifications on chromosome 2p

A total of 237 cases with MNAs (237/628, 37.7%) were detected. Among them, 218 exhibited high-level amplification of *MYCN* ($CN_{MYCN} > 4*CN_{BG}$), and 19 had low-level amplification ($CN_{MYCN} < 4*CN_{BG}$). In addition to MNA on chromosome 2p, 25 cases of high-level *ALK* amplification (**Supplementary Fig. 2**) and seven cases of low-level *ALK* amplification were detected [1,6,7]. All of those also presented MNA. Focal amplifications of 2p25.1 and 2p24.1 were also recurrently detected in conjunction with MNA in this study (**Supplementary Fig. 2**). A few previous studies have also provided evidence that these regions were co-amplified with MNA [7-10]. In our study, 2p25.1 amplification was observed in 23 MNA samples (20/237; 9.7%), which generally covered a genomic region from gene *HPCAL1* to *GREB1*. However, in most of the cases (15/20), only part of this region were amplified. Amplification of the consecutive genes *HPCAL1*-*ODC1*-*NOL10* and *ROCK2*-*E2F6*-*GREB1* was observed in most cases (**Supplementary Table 2**). Overexpressed *ODC1* was associated with reduced survival in neuroblastoma patients, and has been reported as a critical determinant of *MYCN* oncogenesis [11]. *ROCK2*, *E2F6* and *GREB1* may also play an important role in tumor genesis [12-16]. The amplification of 2p24.1 (4/237, 1.7%) was also described previously [7,8]. One of the four samples with 2p24.1 amplification has mRNA expression data, and only *HS1BP3* was highly overexpressed in the amplified region.

2. Focal amplifications on other genomic regions

***MYC* amplification:** two cases of *MYC* high-level amplification were detected including one case showing both *MYC* and *ZFHX3* high-level amplification (**Supplementary Fig. 3**). Two cases of low-level *MYC* amplification were detected including one case showing both *MYC* and *ZFHX3* amplification found another case showing both *MYC* and *ZFHX3* low-level amplification. None of the four cases was observed with MNA. Amplification of *MYC* and *ZFHX3* (*ATBF1*) has been reported in the SJNB12 cell line, but *MYC* amplification has not been observed in neuroblastoma primary tumors before [7,17]. A previous study also reported that chromothripsis resulted in the amplification (low-level) of *MYC* in one case [18]. In addition, *MYC* was highly expressed rather than *MYCN* in Neuroblastoma-derived cell lines lacking amplified *MYCN* [19,20]. Here, our results showed *MYC* amplification existed not only in cell lines but also in neuroblastoma primary tumors, and suggest that *MYC* amplification may be a rare alternative mechanism instead of MNA in neuroblastoma carcinogenesis.

***ZFHX3* (*ATBF1*) amplification:** two cases of *ZFHX3* high-level amplification were detected including one case showing both *MYC* and *ZFHX3* high-level amplification. Seven cases were found with low-level *ZFHX3* amplifications and only one of them exhibited low-level MNA. *ZFHX3* has been reported to function as a tumor suppressor in several cancers [21,22]. *ZFHX3* also plays a role in multiple other biological processes that are regulated by progesterone-PR, including cell proliferation, cell differentiation and tumorigenesis in the mammary gland [23].

***KRAS* amplification:** two cases were found (**Supplementary Fig. 3)**. One case exhibited MNA and another case displayed *MDM2* amplification. As a member of *RAS* oncogene family, *KRAS* amplification has been reported in a variety of different cancers [24-26]. Moreover, a recent study implicated recurrent new mutations of *KRAS* in relapse neuroblastomas [27].

***RRAS2* amplification:** one case was found (**Supplementary Fig. 3)**. *RRAS2* amplification was also detected in the cell line, CHLA-136.

***CYTH1* amplification:** two cases were found and both displayed MNA. Members of this family appear to mediate the regulation of protein sorting and membrane trafficking. This gene is highly

expressed in natural killer and peripheral T cells, and regulates the adhesiveness of integrins at the plasma membrane of lymphocytes. The encoded protein is 83% homologous to that of *CYTH2* (provided by RefSeq, Aug 2008).

***MDM2* amplification:** five cases were detected. Only one case displayed MNA (**Supplementary Fig. 3**). *MDM2* amplification was also detected in cell lines, including NGP and SMS-KAN (both cell lines exhibited MNA and 11q deletion). *MDM2* (*MDM2* oncogene, E3 ubiquitin protein ligase) can promote tumor formation by targeting tumor suppressors, such as p53, for proteasomal degradation. This gene is itself transcriptionally-regulated by p53. *MDM2* amplification has been reported previously in neuroblastoma as a rare event [4,28-31].

***CCND1* amplification:** two cases of high-level amplification were detected. Both cases showed MNA (**Supplementary Fig. 3**). *CCND1* amplification has been reported in diverse tumor types [32,33], and has been reported as a rare event in neuroblastoma[2,7,34]. However, besides the two high-level amplification of *CCND1*, we found 56 cases with low-level *CCND1* amplification (56/628, 8.9%) in our study. Moreover, we found low-level *CCND1* amplification co-occurred with 11q deletion in most cases (53/56, 94.6%, **Supplementary Data 1 and Fig. 3**).

19q13.42 amplification: seven cases were found. The 19q13.42 region contains a cluster of microRNA coding genes (C19MC) [5,35,36], and its amplification appears to be present in pediatric embryonal tumors with multilayered rosettes including Embryonal tumors with abundant neuropil and true rosettes (ependymoblastoma and ETANTR) [35,37-39]. Histologically, ETANTR combines the features of a neuroblastoma and an ependymoblastoma, by showing fine fibrillary neuropil-like areas admixed with cellular regions and ependymoblastoma-like rosettes [40]. Accordingly, in our study, the seven cases with 19q13.42 amplification also show the genetic feathers of neuroblastoma such as MNA, *MDM2* amplification, 17p gain and 1p deletion. None of them was observed with 11q deletion. Interesting, three of the seven cases displayed evidence of chromothripsis in chromosome 19, suggesting chromothripsis may be an important cause of 19q13.42 amplification.

***LIN28B* amplification:** one case was found that displayed MNA. Three cases were identified with low-level *HACE1-LIN28B* amplification. Among them, one exhibited MNA. Common variants within *HACE1-LIN28B* locus have been shown to contribute to neuroblastoma susceptibility. Significant growth inhibition was observed upon depletion of *LIN28B* in neuroblastoma cell lines, and low *HACE1* and high *LIN28B* expression were associated with worse overall survival in neuroblastomas [41]. The *LIN28B* amplification has been reported previously as a rare event [3].

3. Focal amplifications detected in cell lines

***MYCM+ALK+MEIS1+ANTXR1* amplification**: *MYCN* and *ALK* were co-amplified in three cell lines, CHLA-100, IMR5 and IMR32. Interestingly, all of them also exhibited focal amplifications of *MEIS1* and *ANTXR1*, which were not detected in tumors. *MEIS1* is a homeobox gene and has been associated with many cancers. *MEIS1* amplification was also detected in IMR32 previously [42-44]. *ANTXR1* also involves in cell attachment and migration, and has been associated with many cancers [45-47].

Other focal amplifications: other focal amplifications include *MDM2* and *CDK4* co-amplification in NGP, SMS-KAN and *RRAS* amplification in CHLA-136, *MET* amplification in CHP-134, and focal amplification of *GBP2*, *GBP4*, *GBP5* and *GBP7* in CHP-134 and SMS-KCNR.

4. Focal amplifications confirmed by gene expression data

Amplifications of chromosome 2p: three tumor samples and two cell lines with *ALK* amplification have mRNA expression data. The expression levels of *ALK* in the three tumor samples with *ALK* amplification were much higher than the tumor samples without *ALK* amplification. Though much lower than in tumor samples with *ALK* amplification, the expression levels of *ALK* in two cell lines with *ALK* amplification were also higher (**Supplementary Fig. 4**). Among the three tumor samples with *ALK* amplification, X1581425224_A displayed amplifications of 2p25.1 (*ROCK2-E2F6-GREB1*) and 2p24.1 (*HS1BP3*). X1800835432_A displayed amplification of 2p25.1 (*ODC1*). For the amplified region of sample X1581425224_A on 2p25.1, *ROCK2* and *GREB1* showed an elevated expression level of mRNAs, whereas *E2F6* did not. For the amplified region of sample X1581425224_A on 2p24.1 (chr2:20,551,993-21,505,938), only *HS1BP3* was

overexpressed. For the amplified region of X1800835432_A on 2p25.1, only *ODC1* was overexpressed (**Supplementary Fig. 4**).

***CCND1* amplification (low level)**: six tumor samples with low *CCND1* amplification have mRNA expression data. The expression level of *CCND* in the six tumor samples was much higher than other tumor samples and the cell line samples (**Supplementary Fig. 4**).

***MYC* amplification**: one tumor sample with *MYC* amplification has mRNA expression data. The expression of *MYC* in this tumor sample was much higher than other tumor samples and the cell line samples (**Supplementary Fig. 4**).

***LIN28B* amplification**: one tumor sample with *LIN28B* amplification has mRNA expression data. The expression of *LIN28B* in this tumor sample was significantly higher compare to other tumor samples and the cell line samples (**Supplementary Fig. 4**).

***CYTH1* amplification**: one tumor sample with *CYTH1* amplification has mRNA expression data. The amplified region is chr17, 76,509,989-76,758,251 including *DNAH17* and *CYTH1*, however, only the expression of *CYTH1* in this tumor sample was significantly higher than other tumor samples and the cell line samples (**Supplementary Fig. 4**).

Focal amplifications in Cell lines: the amplifications of *ALK*, *MEIS1*, *ANTXR1*, *MDM2*, *CDK4* and *FOXN4* in cell lines, were also confirmed by expression data (**Supplementary Fig. 4**).

5. Focal deletions

3p21.3 deletion: 3p21.3 deletion has been reported in many cancers including neuroblastoma [48-50]. Here, we found four tumors and three cell lines (NGP, NMB, SMS-KAN) with 3p21.3 deletion (**Supplementary Fig. 5**). All of them exhibited 11q deletion. It has been reported that 3p loss is associated with 11q loss, suggesting that the tumor suppressors gene located in 3p21.3 and 11q may interact with each other in neuroblastoma [51,52].

9p21.3 deletion: 9p21.3 deletion, which occurs in many cancers, has been reported as an uncommon event in neuroblastoma [53-61] (**Supplementary Fig. 5**). Genes located in this region are *MTAP*, *CDKN2A* and *CDKN2B*. In this study, 26 cases of 9p21.3 deletion have been detected.

***PTPRD* microdeletion:** Stallings et al first reported the *PTPRD* microdeletion of neuroblastoma in three tumors and three cell lines by array CGH, and Molenaar et al further confirmed this *PTPRD* defect by whole genome sequencing [18,30]. Furthermore, Meehan et al suggested that *PTPRD* plays a tumor suppressor role through AURKA dephosphorylation and destabilization and a downstream destabilization of *N-MYC* in neuroblastoma [62]. In this study, we found 17 tumors with *PTPRD* microdeletion (**Supplementary Fig. 6**). 14 of them exhibited 11q deletion, and only one of them displayed MNA. Besides the previously reported cell lines (KELLY, NGP, SK-N-AS) [30], we also found *PTPRD* microdeletion in cell lines CHP-134, CHP-212, NMB, SMS-KCNR.

6. Chromothripsis

Chromothripsis is a shredding of chromosomal regions and subsequent random reassembly of the fragments, which leads to multiple segmental Gains and/or Losses including loss of tumor suppressors and oncogene amplifications [63,64]. A previous study suggested that the neuroblastoma with chromothripsis were associated with poor prognosis [18]. While chromothripsis was typically identified by whole genome sequencing [18], several hallmarks of chromothripsis can be observed in our study such as alternating copy number states, loss of heterozygosity and high level of breakpoints within confined chromosomal regions [65,66].

In this study, we found 46 (45/628, 7.2%) tumors showing strong evidence of genomic catastrophes with 26 of them (41/46, 89.1%) having MNA or 11q deletion (**Supplementary Fig. 7**). 13 tumors (13/45, 28.9%) have undergone chromothripsis in chromosome 5, including 5p. The frequency is very similar with the previous study of Molenaar et al (3/10, 30%) [18]. 12 tumors (12/45, 26.7%) have undergone chromothripsis in chromosome 2, including 2p. All of them show MNA, and seven of them show *ALK* amplification, suggesting that chromothripsis in chromosome 2 may result in the amplification of *MYCN* and *ALK*. Other chromosomes undergoing chromothripsis

includes chromosomal 1, 6, 7, 11, 12, 13, 17, 18, 19. Interestingly, chromothripsis in chromosome 19 may lead to the amplification of 19q13.42.

a

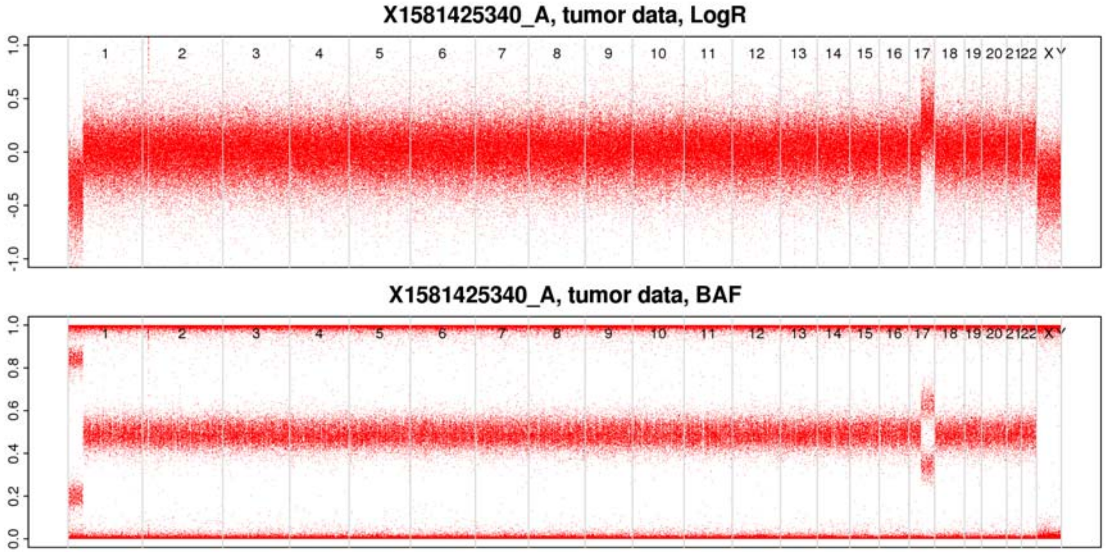

b

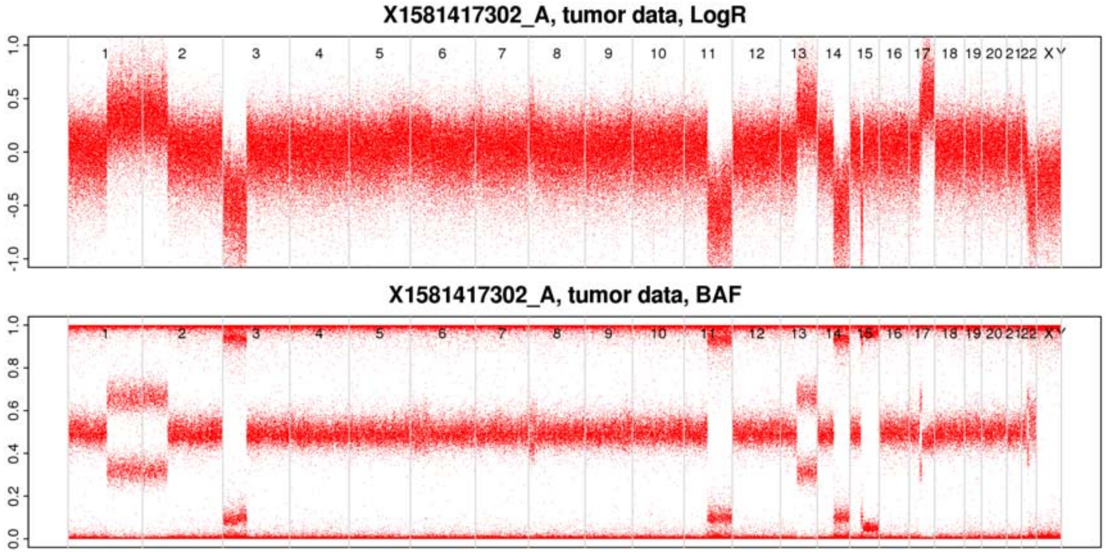

Supplementary Figure 1. BAF and LRR plots of two representative samples of *MYCN* amplification and 11q deletion. (a) *MYCN* amplification with 1p deletion and 17q gain. **(b)** 11q deletion with 17q gain.

a

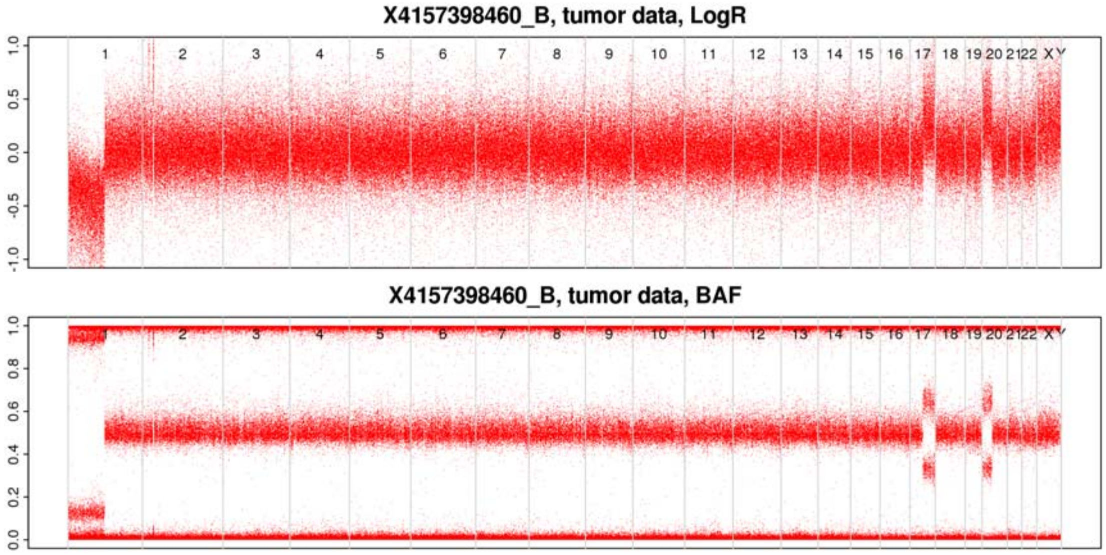

b

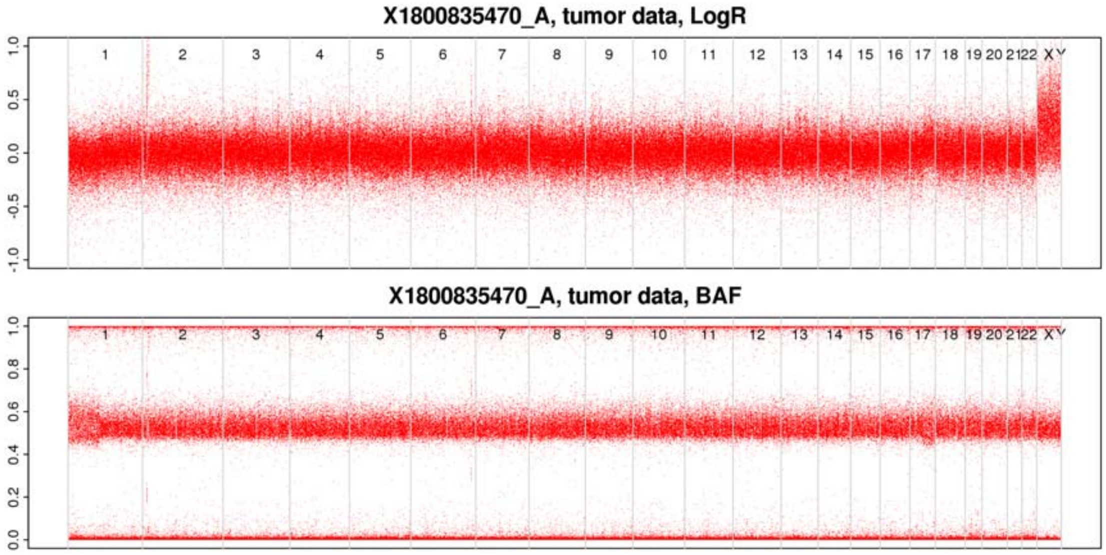

Supplementary Figure 2. BAF and LRR plots of representative samples of *ALK* amplification and 2p25.1 amplification. (a) *ALK* + *MYCN* amplification (two focal regions with significantly elevated LRR value on chr 2 p). **(b)** 2p25.1 amplification with *MYCN* amplification (2p25.1 is very close to *MYCN* (2p24.1)).

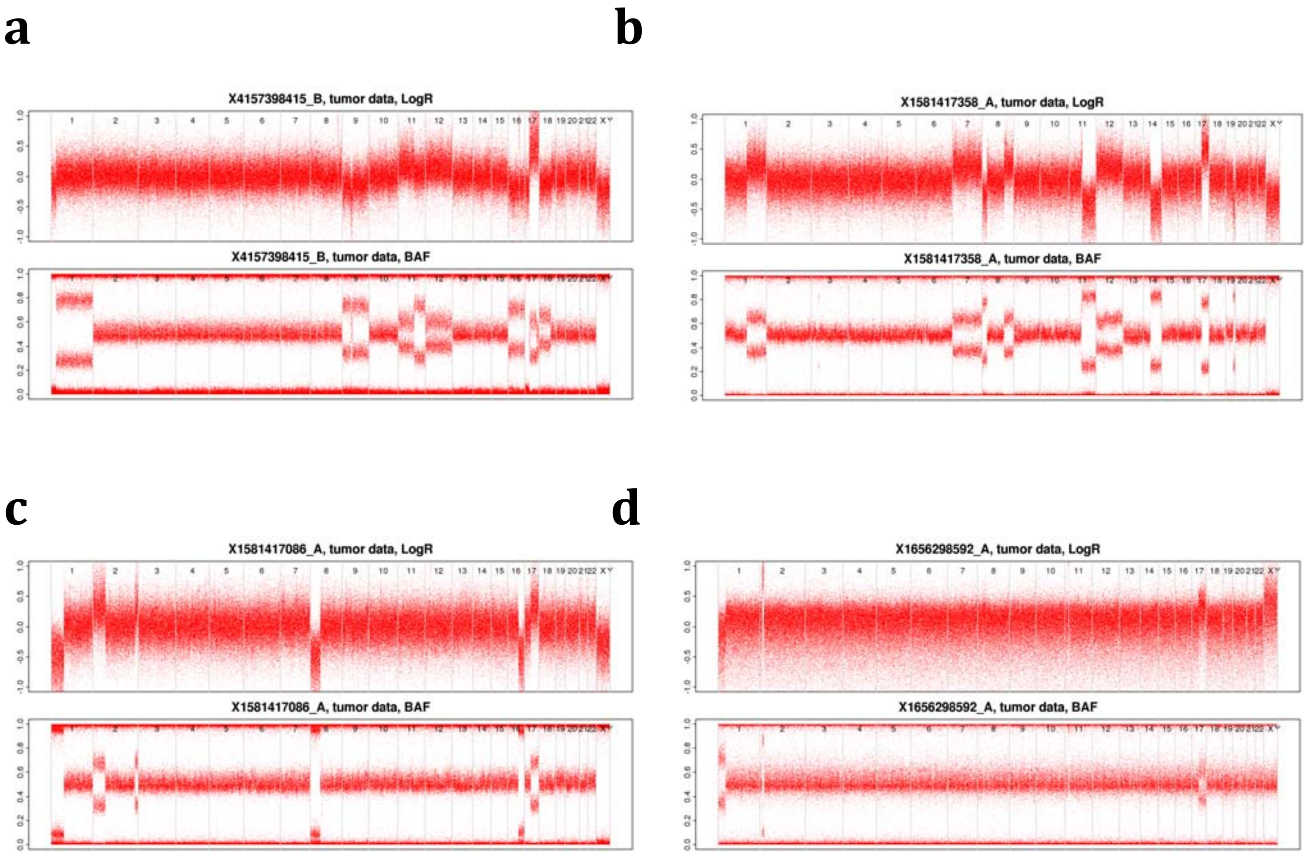

Supplementary Figure 3. BAF and LRR plots of four representative samples on genomic regions other than 2p. (a) *MYC*+*ZFHX3* amplification (*MYC* amplification on chr 8, *ZFHX3* amplification on chr 16). **(b)** Co-occurrence of low-level *CCND1* amplification and 11q deletion. **(c)** *KRAS* amplification (chr 12). **(d)** *RRAS2* amplification (chr 11).

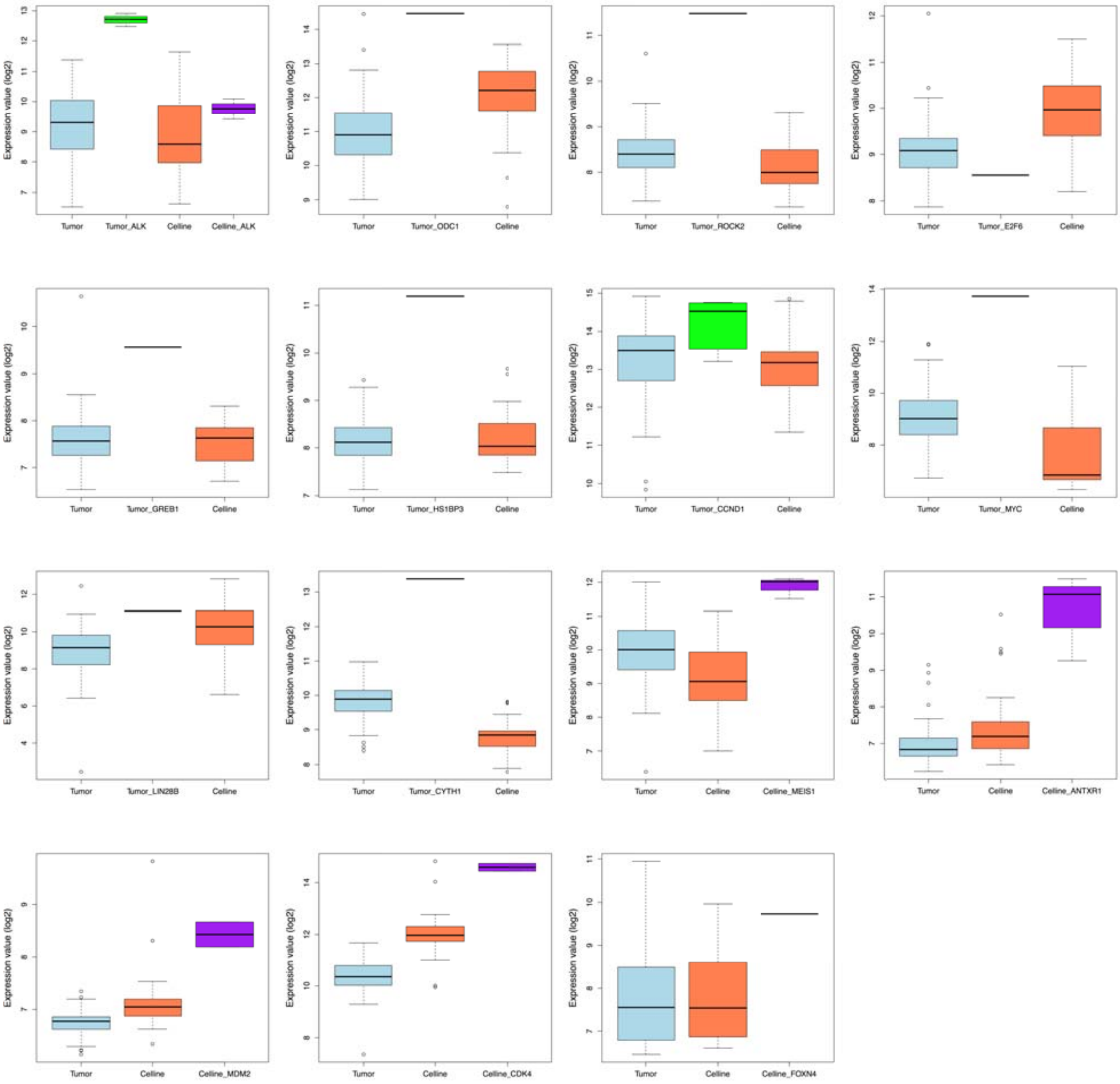

Supplementary Figure 4. mRNA expression levels of amplified genes detected by SNP array. (blue box, tumor samples without detection of targeted gene amplification/ green box, tumor samples with detection of targeted gene amplification/ coral box, cell line samples without detection of targeted gene amplification/ purple box, cell line samples with detection of targeted gene amplification). Boxes in boxplots represent first to third quartiles and whiskers extend to furthest data point still within 1.5 IQRs of either quartile. Center lines in boxes represent medians.

a

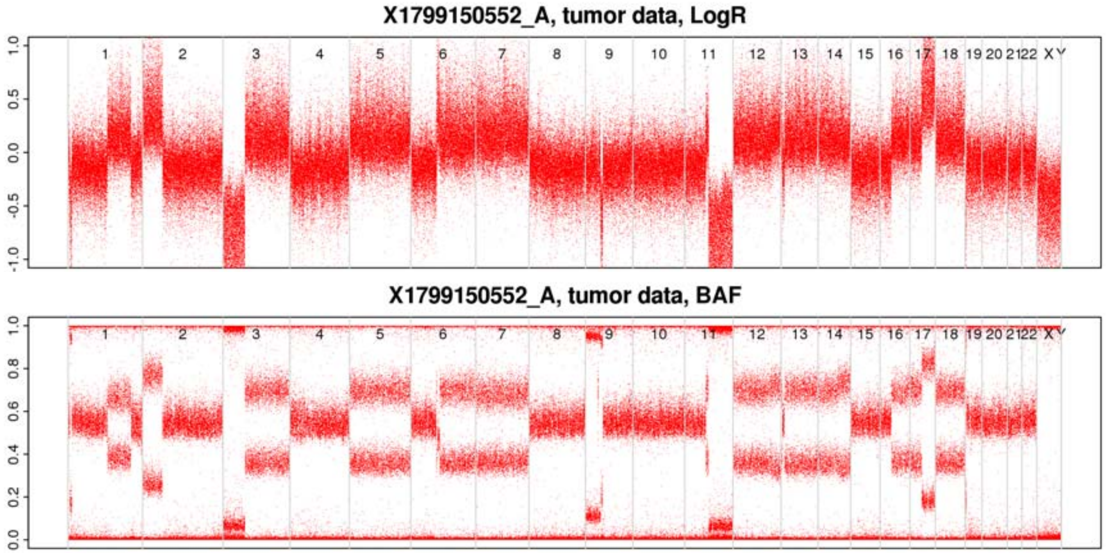

b

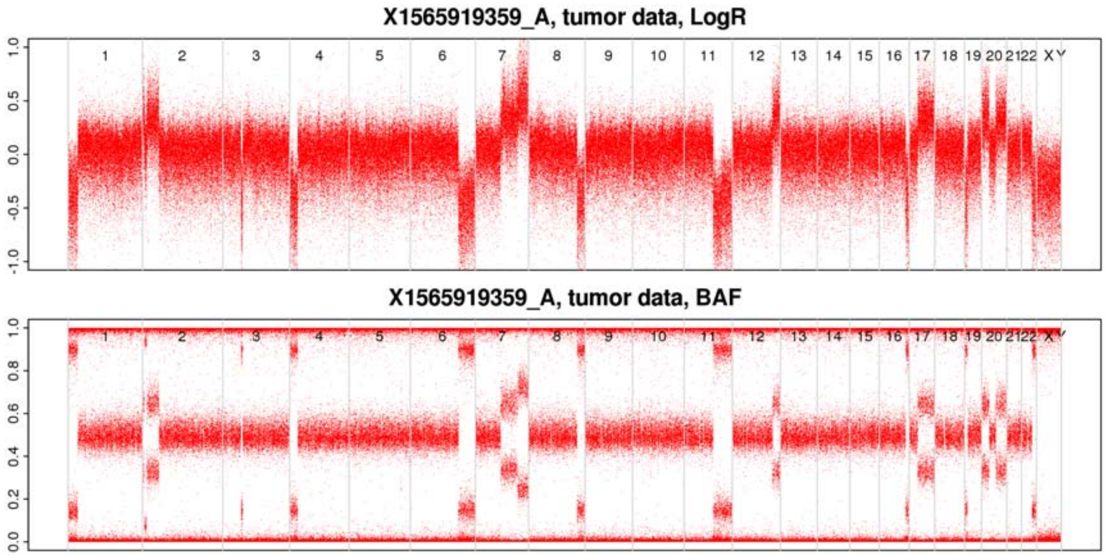

Supplementary Figure 5. BAF and LRR plots of two representative samples of focal deletion. **(a)** 9p21.3 deletion. **(b)** 3p21.3 deletion.

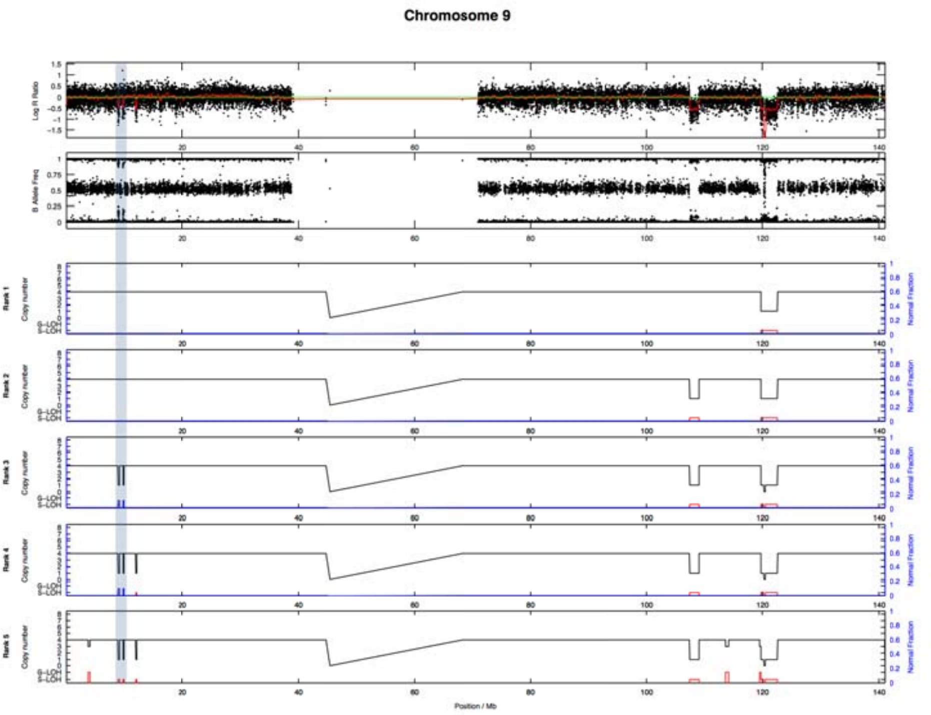

Supplementary Figure 6. BAF and LRR plots of a representative case of *PTPRD* microdeletion (plots generated by OncoSNP).

a

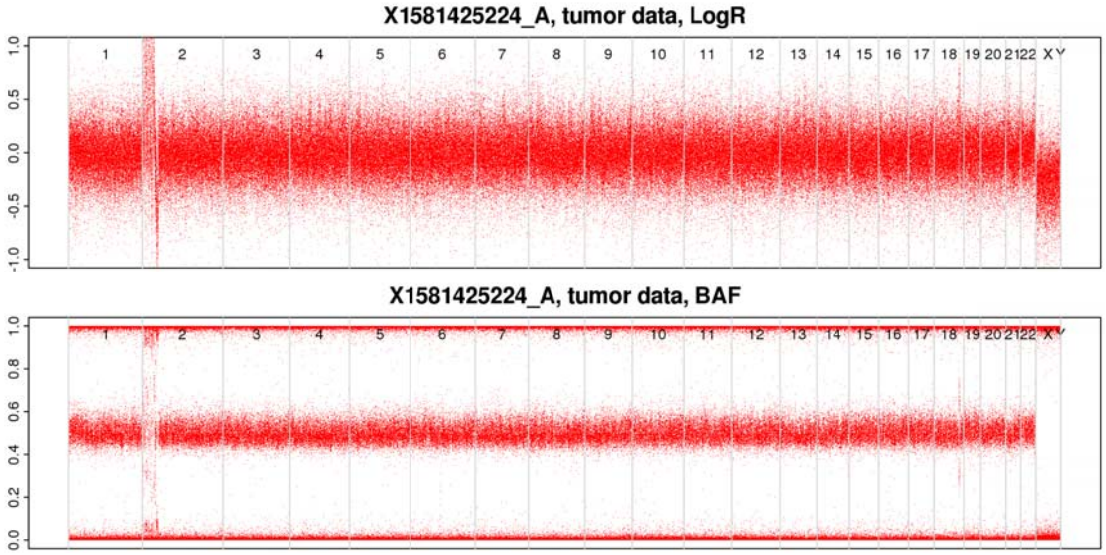
X1581425224_A, tumor data, LogR
X1581425224_A, tumor data, BAF

b

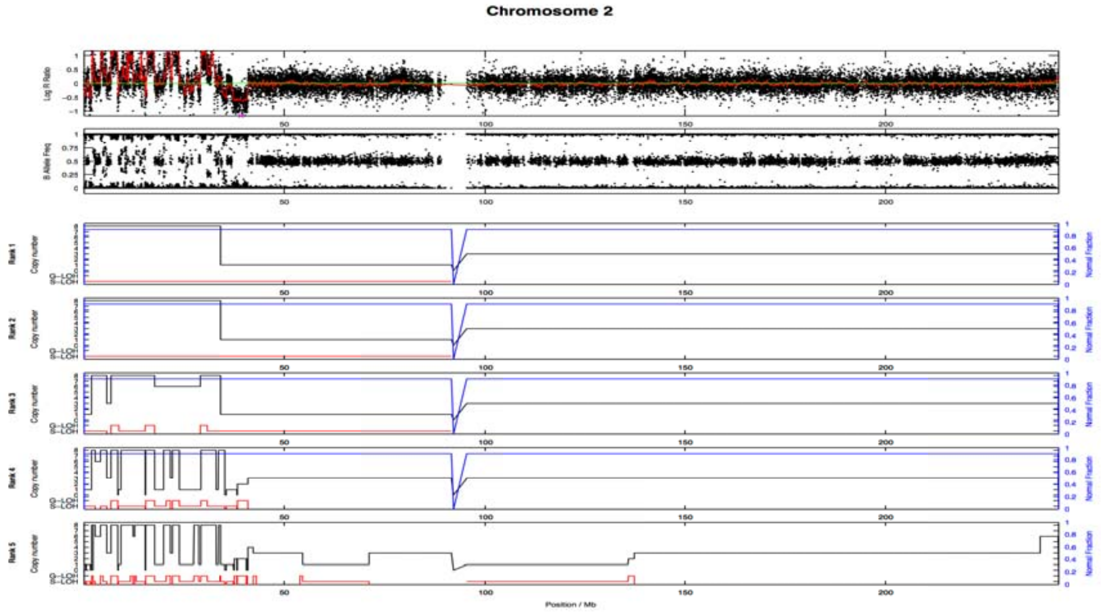
Chromosome 2
Position / Mb

Supplementary Figure 7. BAF and LRR plot of a representative case undergoing chromothripsis in chromosome 2. (a) BAF/LRR plot of the whole genome. **(b)** BAF/LRR plot of chromosome 2 only.

a **b**

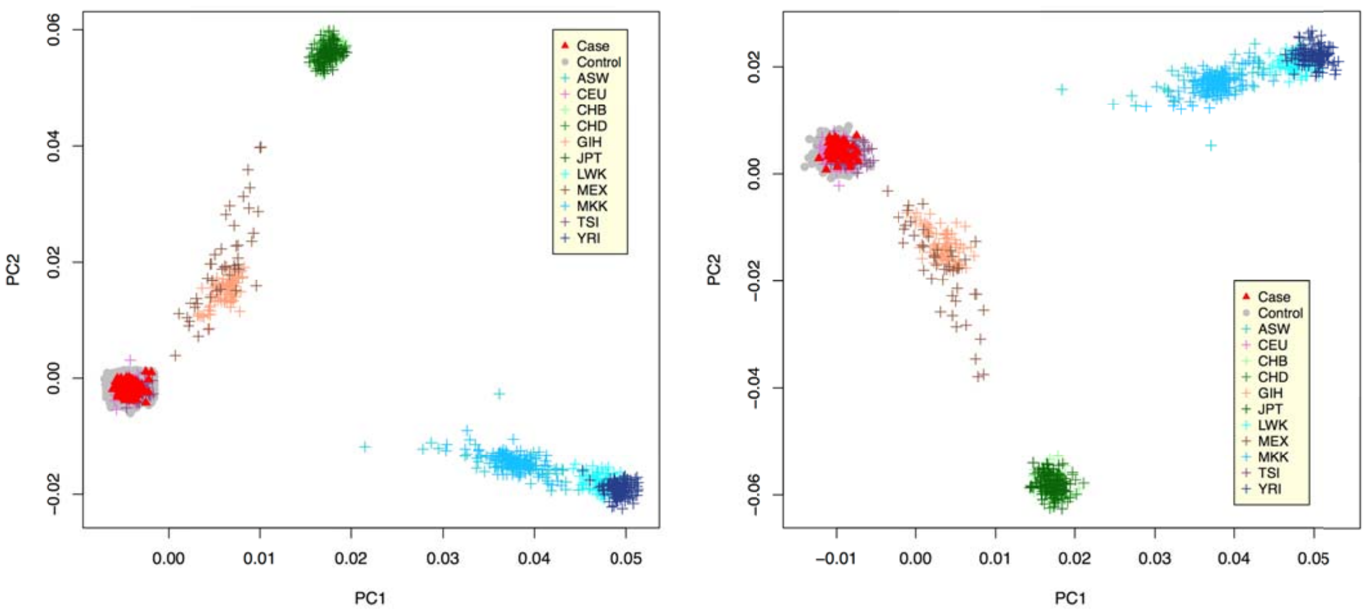

Supplementary Figure 8. Plot of HapMap3 populations and 11q-deletion neuroblastoma cases and controls of European-American individuals used in this study. (a) Discovery study. **(b)** Replication study.

a

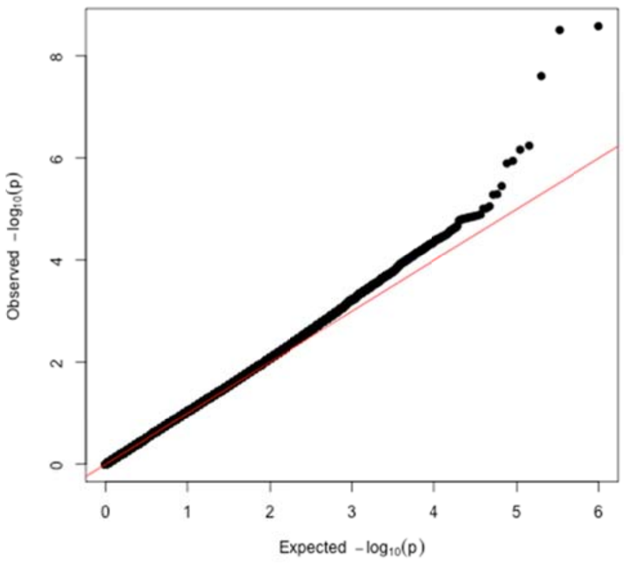
Observed −log10(p)
Expected −log10(p)

b

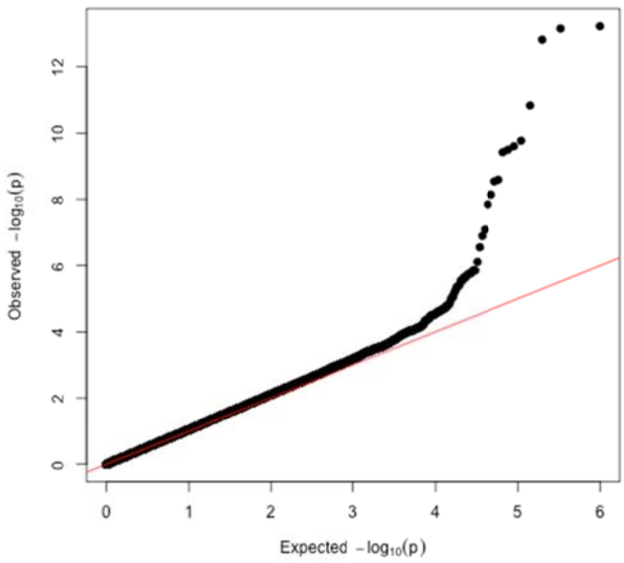
Observed −log10(p)
Expected −log10(p)

c

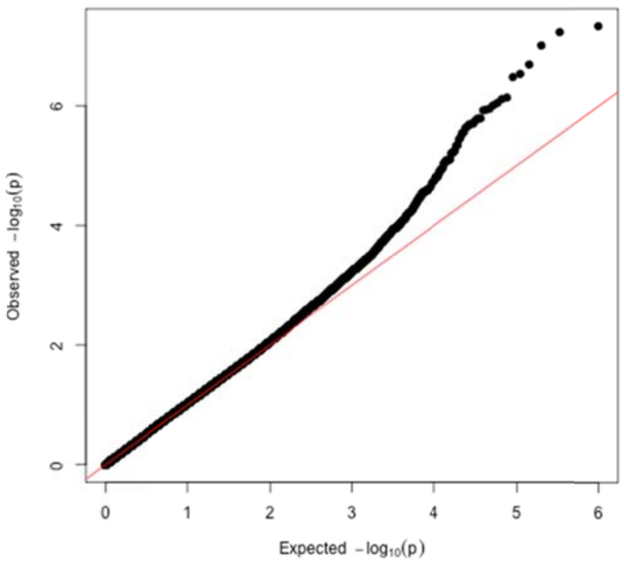
Observed −log10(p)
Expected −log10(p)

Supplementary Figure 9. Quantile-Quantile plot of the expected and observed *P*-values. SNPs passing quality control are plotted. **(a)** 11q-deletion GWAS. **(b)** MNA GWAS. **(c)** 1p-deletion GWAS.

a

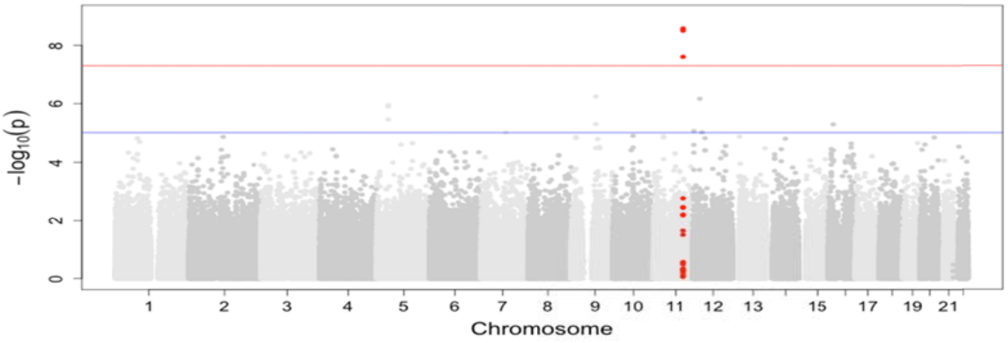

b

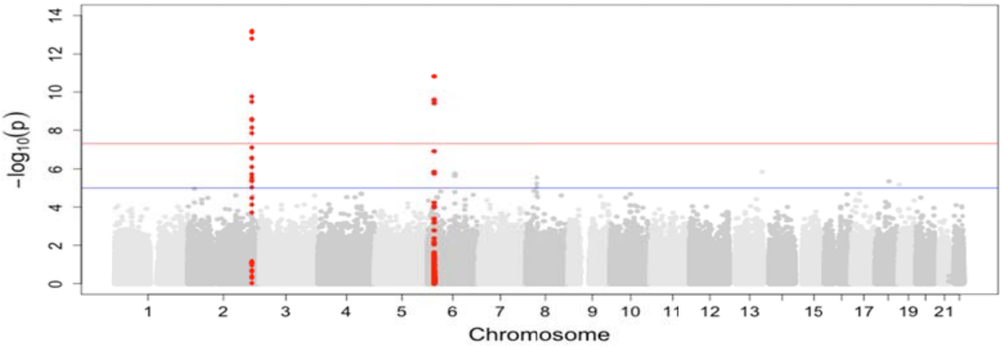

c

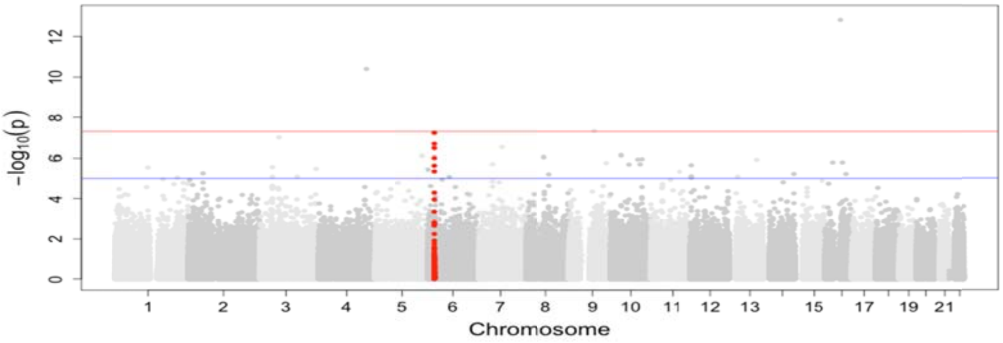

Supplementary Figure 10. Manhattan plots of 11q-deletion GWAS, MNA GWAS and 1p-deletion GWAS. The red and blue horizontal line represents genome-wide significance threshold and suggestive significance threshold respectively. (A) 11q-deletion GWAS, SNPs at the 11q22.2 (*MMP20*) are colored in red. (B) MNA GWAS, SNPs at the 2q35 (*BARD1*) and 6p22.3 (*CASC15*) are

colored in red. (C) 1p-deletion GWAS, SNPs at 6p22.3 (*CASC15*) are colored in red. **(a)** 11q-deletion GWAS. **(b)** MNA GWAS. **(c)** 1p-deletion GWAS.

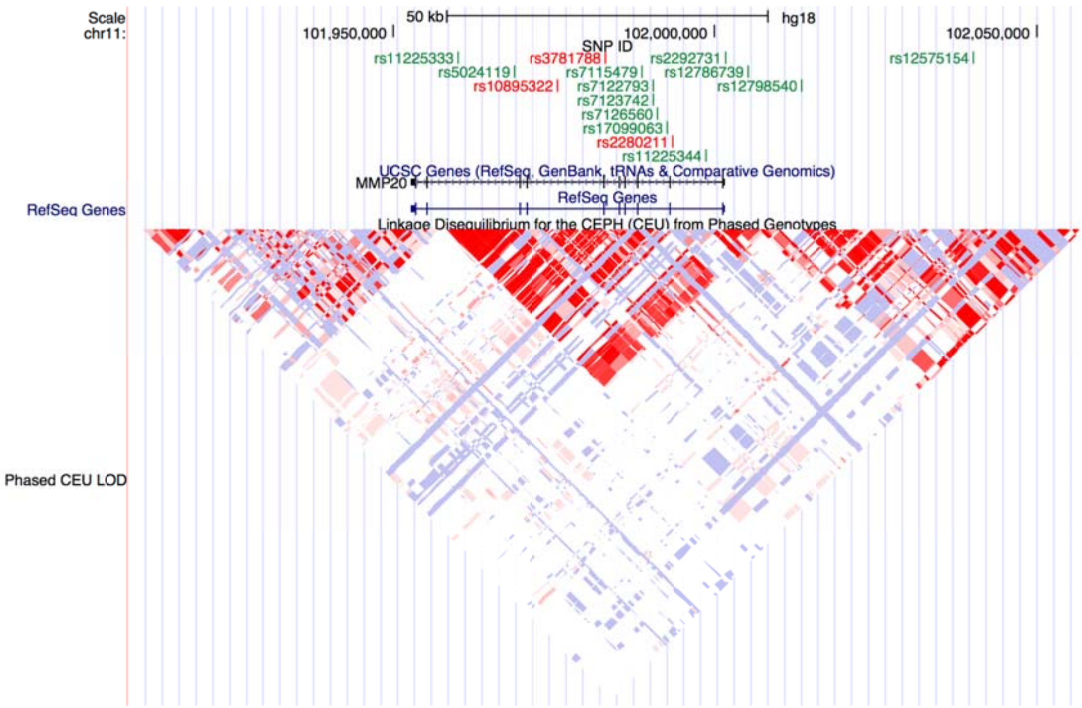

Supplementary Figure 11. UCSC Genome Browser LOD plot of *MMP20*. SNPs reached genome-wide significance in overall meta-analysis of the combined discovery and replication cohorts are plotted, the genotyped SNPs are marked in red, imputed SNPs are marked in green.

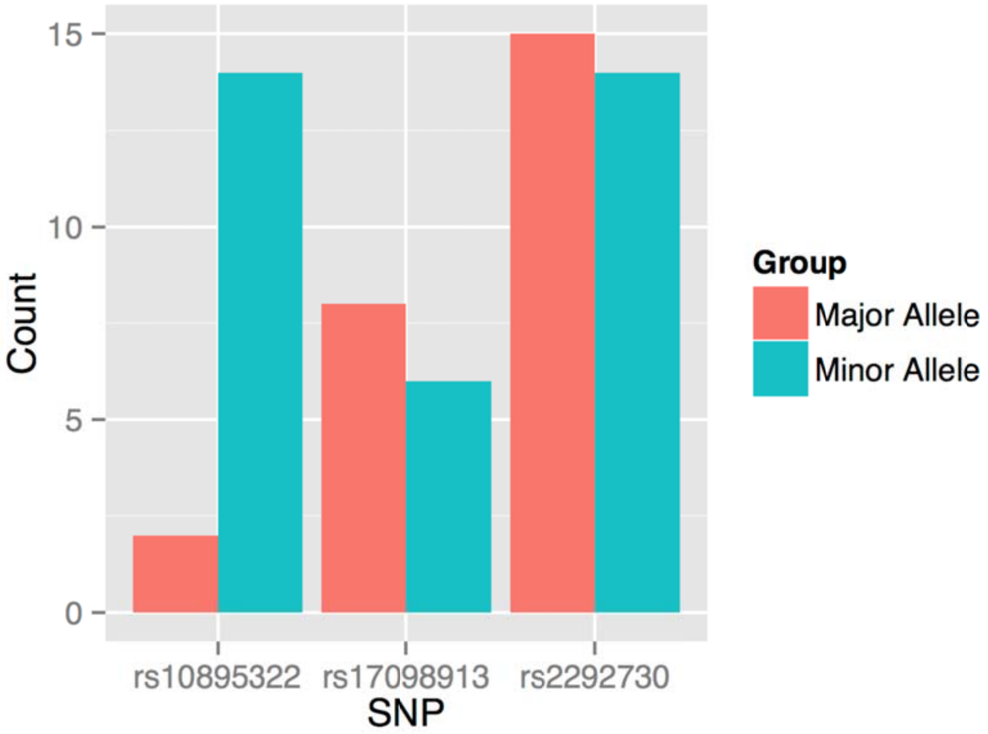

Supplementary Figure 12. Preferential allelic imbalance of rs10895322 in 11q-deletion neuroblastoma. rs10895322 is the top SNP identified in the 11q-deletion GWAS, the risk allele of rs10895322 is the minor allele. rs17098913 and rs2292730 are two SNPs located very close to rs10895322, but show no association with the risk of 11q-deletion neuroblastoma. Here, the minor allele (risk allele) of rs10895322 is significantly retained in the tumor (P = 2.09 × 10^{-3}, while minor alleles of rs17098913 and rs2292730 are randomly lost in tumors (P = 0.39 and P = 0.48 respectively).

Supplementary Table 1. Significance of differential gene expression between CN-amplification samples and no-amplification samples.

Gene	Type	n1/n2	P
ALK	Tumor	3/97	9.33E-07
ALK	Celline	3/35	0.007
CCND1	Tumor	6/94	0.014
MYC	Tumor	1/99	< 2.2E-16
LIN28B	Tumor	1/99	< 2.2E-16
ODC1	Tumor	1/99	< 2.2E-16
ROCK2	Tumor	1/99	< 2.2E-16
E2F6	Tumor	1/99	2.05E-14
GREB1	Tumor	1/99	< 2.2E-16
HS1BP3	Tumor	1/99	< 2.2E-16
CYTH1	Tumor	1/99	< 2.2E-16
MEIS1	Celline	3/35	1.97E-05
ANTXR1	Celline	3/35	0.038
MDM2	Celline	2/36	0.079
CDK4	Celline	2/36	4.28E-4

n1/n2: number of samples with CN amplification/number of samples without CN amplification
P: Student's t-test (one sample t-test was used for n1=1; Welch Two Sample t-test was used for n1>1)

Supplementary Table 2. Recurrently amplified focal regions on chr 2p in samples with MNA. Star sign denotes the gene is partially amplified.

Sample ID			p25.1				p24.1
X1581425402_A	*HPCAL1*	*ODC1*	*NOL10*	*ROCK2*	*E2F6*	*GREB1*	
X1552034382_A	*HPCAL1*	*ODC1*					
X1552034339_A							*HS1BP3*
X1581417155_A							*HS1BP3*
X1552034137_A	*HPCAL1*	*ODC1*	*NOL10*				
X1581994342_A			*NOL10*	*ROCK2*	*E2F6*		
X1581994579_A	*HPCAL1*	*ODC1*	*NOL10*				*HS1BP3*
X1581425084_A	*HPCAL1*	*ODC1*		*ROCK2**	*E2F6*	*GREB1**	
X1581425224_A			*NOL10**	*ROCK2*	*E2F6*	*GREB1*	*HS1BP3*
X1800835432_A	*HPCAL1*	*ODC1*	*NOL10*				
X1800835470_A			*NOL10*	*ROCK2*	*E2F6*	*GREB1**	
X1544641163_A	*HPCAL1*	*ODC1*	*NOL10*	*ROCK2*	*E2F6*	*GREB1*	
X1565901521_A	*HPCAL1*	*ODC1*	*NOL10*	*ROCK2*	*E2F6*	*GREB1*	
X1562865280_A	*HPCAL1*	*ODC1*	*NOL10*	*ROCK2*	*E2F6*		
X4157398460_A	*HPCAL1*	*ODC1*					
X4157398166_A	*HPCAL1**	*ODC1*	*NOL10*	*ROCK2*	*E2F6*	*GREB1*	
X4157398355_B			*NOL10*	*ROCK2*	*E2F6*	*GREB1*	
X1581417389_A	*HPCAL1*	*ODC1*	*NOL10*	*ROCK2*	*E2F6*	*GREB1*	
X1565919582_A	*HPCAL1*	*ODC1*			*E2F6*		
X1581425371_A					*E2F6*	*GREB1*	
X1565919524_A				*ROCK2**	*E2F6*		
X1656298592_A	*HPCAL1*	*ODC1*	*NOL10*	*ROCK2*	*E2F6*	*GREB1*	
X3999080149_R10C02	*HPCAL1**	*ODC1*	*NOL10*	*ROCK2*	*E2F6*	*GREB1*	
X3999081045_R10C02	*HPCAL1*	*ODC1*	*NOL10**				
X3999081076_R03C01	*HPCAL1**	*ODC1*	*NOL10**				

Supplementary Table 3. Association results of neuroblastoma risk loci.

SNP	A1/A2	11q-del GWAS		MNA GWAS		1p-del GWAS	
		OR	***P***	OR	***P***	OR	***P***
**11q22.2 *MMP20***							
rs10895322	G/A	2.858	2.62E-09	1.058	0.755	1.264	0.458
rs3781788	T/C	2.505	2.46E-08	1.117	0.463	1.328	0.288
rs2280211	C/T	2.604	3.11E-09	1.102	0.522	1.417	0.181
**2q35 *BARD1***							
rs3768716	G/A	1.471	8.67E-03	2.004	7.03E-14	2.035	6.64E-05
rs17487792	T/C	1.450	1.18E-02	2.007	5.99E-14	1.956	1.51E-04
rs7587476	T/C	1.452	9.61E-03	1.969	1.52E-13	1.944	1.53E-04
**6p22.3 *CASC15***							
rs4712653	C/T	1.645	2.92E-04	1.858	1.47E-11	2.674	5.87E-08
rs9295536	A/C	1.690	9.08E-05	1.757	3.78E-10	2.471	2.04E-07
rs6939340	G/A	1.708	1.05E-04	1.783	2.50E-10	2.505	3.31E-07
**11p15.4 *LMO1***							
rs110419	A/G	1.572	8.96E-04	1.160	0.101	1.16	0.911

A1/A2: risk allele/protective allele.

P: *P*-value calculated by logistic regression test.

Association results are reported from the newly discovered *MMP20* locus, and three previously reported loci including *BARD1*, *CASC15*, *LMO1*. (Previously identified gene loci *HACE1-LIN28B* and *HSD17B12* were excluded, as no significant SNP was observed at these loci.)

Supplementary Table 4. Pairwise interaction between most significant SNPs at four risk loci (case/control).

CHR1	SNP1	Gene1	CHR2	SNP2	Gene2	OR	*P*	*P*-adj
**2**	rs3768716	*BARD1*	6	rs4712653	*CASC15*	1.262	0.286	1
**2**	rs3768716	*BARD1*	11	rs110419	*LMO1*	1.093	0.675	1
**2**	rs3768716	*BARD1*	11	rs10895322	*MMP20*	0.634	0.122	0.732
**6**	rs4712653	*CASC15*	11	rs110419	*LMO1*	0.869	0.485	1
**6**	rs4712653	*CASC15*	11	rs10895322	*MMP20*	0.872	0.620	1
**11**	rs110419	*LMO1*	11	rs10895322	*MMP20*	0.792	0.362	1

P: *P*-value from –-epistasis option in PLINK.

P-adj: *P*-value adjusted by Bonferroni correction.

Supplementary Table 5. Pairwise interaction between most significant SNPs at four risk loci (case-only).

CHR1	SNP1	Gene1	CHR2	SNP2	Gene2	*P*	*P*-adj
**2**	rs3768716	*BARD1*	6	rs4712653	*CASC15*	0.193	1
**2**	rs3768716	*BARD1*	11	rs110419	*LMO1*	0.234	1
**2**	rs3768716	*BARD1*	11	rs10895322	*MMP20*	0.163	0.978
**6**	rs4712653	*CASC15*	11	rs110419	*LMO1*	0.471	1
**6**	rs4712653	*CASC15*	11	rs10895322	*MMP20*	0.611	1
**11**	rs110419	*LMO1*	11	rs10895322	*MMP20*	0.240	1

P: *P*-value from –-fast-epistasis and --case-only options in PLINK.

P-adj: *P*-value adjusted by Bonferroni correction.

Supplementary Table 6. Association results of 11q11.2 (*MMP20*) locus in the GWAS of 113 11q-deletions neuroblastomas and 282 controls (78 11q normal and 204 MNA neuroblastomas).

CHR	SNP	POS	A1	A2	*P*	OR
**11**	rs10895322	102470256	G	A	5.50E-5	2.811
**11**	rs3781788	102477556	T	C	3.92E-4	2.257
**11**	rs2280211	102488131	C	T	7.33E-5	2.474

P: *P*-value calculated by logistic regression test.

Supplementary references

1. Mosse, Y.P. *et al.* Identification of ALK as a major familial neuroblastoma predisposition gene. *Nature* **455**, 930-5 (2008).
2. Molenaar, J.J., van Sluis, P., Boon, K., Versteeg, R. & Caron, H.N. Rearrangements and increased expression of cyclin D1 (CCND1) in neuroblastoma. *Genes Chromosomes Cancer* **36**, 242-9 (2003).
3. Molenaar, J.J. *et al.* LIN28B induces neuroblastoma and enhances MYCN levels via let-7 suppression. *Nat Genet* **44**, 1199-206 (2012).
4. Corvi, R. *et al.* Non-syntenic amplification of MDM2 and MYCN in human neuroblastoma. *Oncogene* **10**, 1081-6 (1995).
5. Li, M. *et al.* Frequent amplification of a chr19q13.41 microRNA polycistron in aggressive primitive neuroectodermal brain tumors. *Cancer Cell* **16**, 533-46 (2009).
6. Osajima-Hakomori, Y. *et al.* Biological role of anaplastic lymphoma kinase in neuroblastoma. *Am J Pathol* **167**, 213-22 (2005).
7. Fix, A. *et al.* Characterization of amplicons in neuroblastoma: high-resolution mapping using DNA microarrays, relationship with outcome, and identification of overexpressed genes. *Genes Chromosomes Cancer* **47**, 819-34 (2008).
8. George, R.E. *et al.* Investigation of co-amplification of the candidate genes ornithine decarboxylase, ribonucleotide reductase, syndecan-1 and a DEAD box gene, DDX1, with N-myc in neuroblastoma. United Kingdom Children's Cancer Study Group. *Oncogene* **12**, 1583-7 (1996).
9. George, R.E. *et al.* Analysis of candidate gene co-amplification with MYCN in neuroblastoma. *Eur J Cancer* **33**, 2037-42 (1997).
10. Beheshti, B. *et al.* Chromosomal localization of DNA amplifications in neuroblastoma tumors using cDNA microarray comparative genomic hybridization. *Neoplasia* **5**, 53-62 (2003).
11. Hogarty, M.D. *et al.* ODC1 is a critical determinant of MYCN oncogenesis and a therapeutic target in neuroblastoma. *Cancer Res* **68**, 9735-45 (2008).
12. Chen, H.Z., Tsai, S.Y. & Leone, G. Emerging roles of E2Fs in cancer: an exit from cell cycle control. *Nat Rev Cancer* **9**, 785-97 (2009).
13. Wang, H.F., Takenaka, K., Nakanishi, A. & Miki, Y. BRCA2 and nucleophosmin coregulate centrosome amplification and form a complex with the Rho effector kinase ROCK2. *Cancer Res* **71**, 68-77 (2011).
14. Liu, M., Wang, G., Gomez-Fernandez, C.R. & Guo, S. GREB1 functions as a growth promoter and is modulated by IL6/STAT3 in breast cancer. *PLoS One* **7**, e46410 (2012).
15. Mohammed, H. *et al.* Endogenous purification reveals GREB1 as a key estrogen receptor regulatory factor. *Cell Rep* **3**, 342-9 (2013).
16. Morgan-Fisher, M., Couchman, J.R. & Yoneda, A. Phosphorylation and mRNA splicing of collapsin response mediator protein-2 determine inhibition of rho-associated protein kinase (ROCK) II function in carcinoma cell migration and invasion. *J Biol Chem* **288**, 31229-40 (2013).
17. Van Roy, N. *et al.* Translocation-excision-deletion-amplification mechanism leading to nonsyntenic coamplification of MYC and ATBF1. *Genes Chromosomes Cancer* **45**, 107-17 (2006).

18. Molenaar, J.J. *et al.* Sequencing of neuroblastoma identifies chromothripsis and defects in neuritogenesis genes. *Nature* **483**, 589-93 (2012).
19. Sadee, W. *et al.* Expression of neurotransmitter receptors and myc protooncogenes in subclones of a human neuroblastoma cell line. *Cancer Res* **47**, 5207-12 (1987).
20. Breit, S. & Schwab, M. Suppression of MYC by high expression of NMYC in human neuroblastoma cells. *J Neurosci Res* **24**, 21-8 (1989).
21. Sun, X. *et al.* Frequent somatic mutations of the transcription factor ATBF1 in human prostate cancer. *Nat Genet* **37**, 407-12 (2005).
22. Sun, X. *et al.* Infrequent mutation of ATBF1 in human breast cancer. *J Cancer Res Clin Oncol* **133**, 103-5 (2007).
23. Li, M. *et al.* Upregulation of ATBF1 by progesterone-PR signaling and its functional implication in mammary epithelial cells. *Biochem Biophys Res Commun* **430**, 358-63 (2013).
24. Birkeland, E. *et al.* KRAS gene amplification and overexpression but not mutation associates with aggressive and metastatic endometrial cancer. *Br J Cancer* **107**, 1997-2004 (2012).
25. Rahman, M.T. *et al.* KRAS and MAPK1 Gene Amplification in Type II Ovarian Carcinomas. *Int J Mol Sci* **14**, 13748-62 (2013).
26. Valtorta, E. *et al.* KRAS gene amplification in colorectal cancer and impact on response to EGFR-targeted therapy. *Int J Cancer* **133**, 1259-65 (2013).
27. Schramm, A. *et al.* Mutational dynamics between primary and relapse neuroblastomas. *Nat Genet* **47**, 872-7 (2015).
28. Corvi, R., Savelyeva, L., Amler, L., Handgretinger, R. & Schwab, M. Cytogenetic evolution of MYCN and MDM2 amplification in the neuroblastoma LS tumour and its cell line. *Eur J Cancer* **31A**, 520-3 (1995).
29. Guimier, A. *et al.* Clinical characteristics and outcome of patients with neuroblastoma presenting genomic amplification of loci other than MYCN. *PLoS One* **9**, e101990 (2014).
30. Stallings, R.L. *et al.* High-resolution analysis of chromosomal breakpoints and genomic instability identifies PTPRD as a candidate tumor suppressor gene in neuroblastoma. *Cancer Res* **66**, 3673-80 (2006).
31. Wolf, M. *et al.* Array-based gene expression, CGH and tissue data defines a 12q24 gain in neuroblastic tumors with prognostic implication. *BMC Cancer* **10**, 181 (2010).
32. Musgrove, E.A., Caldon, C.E., Barraclough, J., Stone, A. & Sutherland, R.L. Cyclin D as a therapeutic target in cancer. *Nat Rev Cancer* **11**, 558-72 (2011).
33. Roy, P.G. *et al.* High CCND1 amplification identifies a group of poor prognosis women with estrogen receptor positive breast cancer. *Int J Cancer* **127**, 355-60 (2010).
34. Carr, J. *et al.* High-resolution analysis of allelic imbalance in neuroblastoma cell lines by single nucleotide polymorphism arrays. *Cancer Genet Cytogenet* **172**, 127-38 (2007).
35. Nobusawa, S. *et al.* Analysis of chromosome 19q13.42 amplification in embryonal brain tumors with ependymoblastic multilayered rosettes. *Brain Pathol* **22**, 689-97 (2012).
36. Kleinman, C.L. *et al.* Fusion of TTYH1 with the C19MC microRNA cluster drives expression of a brain-specific DNMT3B isoform in the embryonal brain tumor ETMR. *Nat Genet* **46**, 39-44 (2014).
37. Pfister, S. *et al.* Novel genomic amplification targeting the microRNA cluster at 19q13.42 in a pediatric embryonal tumor with abundant neuropil and true rosettes. *Acta Neuropathol* **117**, 457-64 (2009).

38. Korshunov, A. *et al.* Focal genomic amplification at 19q13.42 comprises a powerful diagnostic marker for embryonal tumors with ependymoblastic rosettes. *Acta Neuropathol* **120**, 253-60 (2010).
39. Wang, Y. *et al.* Embryonal tumor with abundant neuropil and true rosettes (ETANTR) with a focal amplification at chromosome 19q13.42 locus: further evidence of two new instances in China. *Neuropathology* **31**, 639-47 (2011).
40. Eberhart, C.G., Brat, D.J., Cohen, K.J. & Burger, P.C. Pediatric neuroblastic brain tumors containing abundant neuropil and true rosettes. *Pediatr Dev Pathol* **3**, 346-52 (2000).
41. Diskin, S.J. *et al.* Common variation at 6q16 within HACE1 and LIN28B influences susceptibility to neuroblastoma. *Nat Genet* **44**, 1126-30 (2012).
42. Jones, T.A., Flomen, R.H., Senger, G., Nizetic, D. & Sheer, D. The homeobox gene MEIS1 is amplified in IMR-32 and highly expressed in other neuroblastoma cell lines. *Eur J Cancer* **36**, 2368-74 (2000).
43. Spieker, N. *et al.* The MEIS1 oncogene is highly expressed in neuroblastoma and amplified in cell line IMR32. *Genomics* **71**, 214-21 (2001).
44. Geerts, D., Schilderink, N., Jorritsma, G. & Versteeg, R. The role of the MEIS homeobox genes in neuroblastoma. *Cancer Lett* **197**, 87-92 (2003).
45. Nanda, A. *et al.* TEM8 interacts with the cleaved C5 domain of collagen alpha 3(VI). *Cancer Res* **64**, 817-20 (2004).
46. Cullen, M. *et al.* Host-derived tumor endothelial marker 8 promotes the growth of melanoma. *Cancer Res* **69**, 6021-6 (2009).
47. Gu, J., Faundez, V. & Werner, E. Endosomal recycling regulates Anthrax Toxin Receptor 1/Tumor Endothelial Marker 8-dependent cell spreading. *Exp Cell Res* **316**, 1946-57 (2010).
48. Lazcoz, P. *et al.* Frequent promoter hypermethylation of RASSF1A and CASP8 in neuroblastoma. *BMC Cancer* **6**, 254 (2006).
49. Hoebeeck, J. *et al.* High resolution tiling-path BAC array deletion mapping suggests commonly involved 3p21-p22 tumor suppressor genes in neuroblastoma and more frequent tumors. *Int J Cancer* **120**, 533-8 (2007).
50. Hesson, L.B., Cooper, W.N. & Latif, F. Evaluation of the 3p21.3 tumour-suppressor gene cluster. *Oncogene* **26**, 7283-301 (2007).
51. Plantaz, D. *et al.* Comparative genomic hybridization (CGH) analysis of stage 4 neuroblastoma reveals high frequency of 11q deletion in tumors lacking MYCN amplification. *Int J Cancer* **91**, 680-6 (2001).
52. Breen, C.J., O'Meara, A., McDermott, M., Mullarkey, M. & Stallings, R.L. Coordinate deletion of chromosome 3p and 11q in neuroblastoma detected by comparative genomic hybridization. *Cancer Genet Cytogenet* **120**, 44-9 (2000).
53. Diaz, M.O. *et al.* Deletions of interferon genes in acute lymphoblastic leukemia. *N Engl J Med* **322**, 77-82 (1990).
54. Einhorn, S., Grander, D., Bjork, O., Brondum-Nielsen, K. & Soderhall, S. Deletion of alpha-, beta-, and omega-interferon genes in malignant cells from children with acute lymphocytic leukemia. *Cancer Res* **50**, 7781-5 (1990).
55. James, C.D., He, J., Collins, V.P., Allalunis-Turner, M.J. & Day, R.S., 3rd. Localization of chromosome 9p homozygous deletions in glioma cell lines with markers constituting a continuous linkage group. *Cancer Res* **53**, 3674-6 (1993).
56. Olopade, O.I. *et al.* Homozygous loss of the interferon genes defines the critical region on 9p that is deleted in lung cancers. *Cancer Res* **53**, 2410-5 (1993).

57. Marshall, B., Isidro, G., Martins, A.G. & Boavida, M.G. Loss of heterozygosity at chromosome 9p21 in primary neuroblastomas: evidence for two deleted regions. *Cancer Genet Cytogenet* **96**, 134-9 (1997).
58. Takita, J. *et al.* Deletion map of chromosome 9 and p16 (CDKN2A) gene alterations in neuroblastoma. *Cancer Res* **57**, 907-12 (1997).
59. Lydiatt, W.M., Davidson, B.J., Schantz, S.P., Caruana, S. & Chaganti, R.S. 9p21 deletion correlates with recurrence in head and neck cancer. *Head Neck* **20**, 113-8 (1998).
60. Giordani, L. *et al.* Two regions of deletion in 9p22- p24 in neuroblastoma are frequently observed in favorable tumors. *Cancer Genet Cytogenet* **135**, 42-7 (2002).
61. Mora, J. *et al.* Comprehensive analysis of the 9p21 region in neuroblastoma suggests a role for genes mapping to 9p21-23 in the biology of favourable stage 4 tumours. *Br J Cancer* **91**, 1112-8 (2004).
62. Meehan, M. *et al.* Protein tyrosine phosphatase receptor delta acts as a neuroblastoma tumor suppressor by destabilizing the aurora kinase A oncogene. *Mol Cancer* **11**, 6 (2012).
63. Stephens, P.J. *et al.* Massive genomic rearrangement acquired in a single catastrophic event during cancer development. *Cell* **144**, 27-40 (2011).
64. Korbel, J.O. & Campbell, P.J. Criteria for inference of chromothripsis in cancer genomes. *Cell* **152**, 1226-36 (2013).
65. Nones, K. *et al.* Genomic catastrophes frequently arise in esophageal adenocarcinoma and drive tumorigenesis. *Nat Commun* **5**, 5224 (2014).
66. Ambros, I.M., Brunner, C., Abbasi, R., Frech, C. & Ambros, P.F. Ultra-High Density SNParray in Neuroblastoma Molecular Diagnostics. *Front Oncol* **4**, 202 (2014).
